# His Bundle Pacing: Predicting Mortality and Major Complications in Mid-Term Follow-Up

**DOI:** 10.3390/jcm13061802

**Published:** 2024-03-21

**Authors:** Piotr Kulesza, Rafał Gardas, Krzysztof S. Gołba, Tomasz Soral, Rafał Sznajder, Grzegorz Jarosiński, Kamil Zub, Danuta Łoboda

**Affiliations:** 1 Department of Electrocardiology and Heart Failure, Medical University of Silesia, 40-007 Katowice, Poland; 2 Department of Electrocardiology, Leszek Giec Upper-Silesian Medical Centre, Silesian Medical University, 40-055 Katowice, Poland; 3 Doctoral School, Medical University of Silesia, 40-007 Katowice, Poland

**Keywords:** His bundle pacing, pacing parameters, complications, safety, mortality and outcome predictors

## Abstract

**Introduction**: His bundle pacing (HBP) is suitable for 80% of patients with any indication for permanent pacemaker implantation, with a clinical benefit compared to right ventricular pacing (RVP). Although complications and mortality related to RVP are widely reported in the literature, data on HBP are limited. This study aimed to analyze HBP complications and outcomes in the short-term (up to 30 days) and long-term (up to the following 24 months) follow-up (F/U). **Materials and Methods**: The study includes 373 patients aged ≥ 18, enrolled from October 2015 to May 2019 in a single-center HBP prospective registry conducted in the Department of Electrocardiology, Upper Silesian Medical Centre of the Medical University of Silesia in Katowice, Poland. Mortality and HBP complications were used as end-points: during hospitalization and up to 30 days (short-term F/U), and for each F/U point—six months, 12 months, and 24 months after the procedure (long-term F/U). **Results**: Successful HBP was achieved in 252 patients (68%), with an increasing success rate during consecutive years: 57% in 2015–2016 and 73% in 2017–2019. Complications were found in 8.4% of patients (21/252) in short-term F/U and 5.8% (13/224), 5.5% (11/201), and 6.9% (12/174) at six months, 12 months, and 24 months, respectively. There were no deaths during the first 30 days. However, 26 patients (10.3%) died within 24 months. A left ventricular ejection fraction (LVEF) ≤ 34% was the only independent predictor of all-cause mortality or any major complication in the 24-month F/U. **Conclusions**: This single-center study reported a low risk of mortality and complications associated with HBP at the short-term F/U. However, during the long-term F/U, we observed a higher but acceptable risk of major complications, with a lower LVEF being an independent predictor of the composite end-point of all-cause mortality or any major complication.

## 1. Introduction

Mortality and complications of classical cardiac implantable electronic device (CIED) treatment are widely reported in the literature [1,2]. It is common knowledge that permanent right ventricular pacing (RVP) induces non-physiological electromechanical myocardial activation, which may lead to structural remodeling of the left ventricle (LV), mitral valve dysfunction, proarrhythmia, and is associated with increased mortality during follow-up (F/U) [1,2]. Chronic RVP is believed to lead to pacing-induced cardiomyopathy, especially in patients with high ventricular pacing burdens [3]. Conduction system pacing (CSP), including His bundle pacing (HBP), is an emerging technique for preventing pacing-related adverse effects [4] and is included in European Society of Cardiology guidelines on cardiac pacing and cardiac resynchronization therapy (CRT) [5]. CSP, initially preferred, is considered safe and suitable in most patients with an indication for pacemaker implantation [2,6]; however, it still has not become the first-choice method of pacing [7]. Studies confirmed HBP’s feasibility, favorable safety profile, and clinical benefits compared with RVP [1,2,8,9]. While there are many reports of RVP or classical CRT complications [10,11,12], data on HBP complications are limited.

This study aimed to analyze all HBP complications and outcomes in the short-term (up to 30 days) and long-term (up to the following 24 months) F/U.

## 2. Materials and Methods

### 2.1. Patients

Data were extracted from a single-center prospective registry of CSP started in October 2015 in the Department of Electrocardiology of Professor Leszek Giec Upper Silesian Medical Centre of the Medical University of Silesia in Katowice, Poland. There were 373 patients aged 18 or older who underwent an attempt to conduct an HBP implantation and who were enrolled from the start of the registry to 15 May 2019.

The commonly accepted indications for HBP were applied as follows:

1. A complete or high-degree atrioventricular (AV) block in the sinus rhythm or atrial fibrillation (AF), especially in patients with a reduced left ventricular ejection fraction (LVEF).
2. Sick sinus syndrome with PR interval > 200 ms, as above, primarily in patients with a reduced LVEF.
3. CRT in patients with an LVEF ≤ 35% [5] and a left or right bundle branch block.
4. Pacing-induced cardiomyopathy (PICM)—defined as congestive heart failure worsening, accompanied by a decline in LVEF < 50% with an RVP burden of ≥40% [13]. HBP was used as the method of choice in these settings, regardless of the device used. HBP was used more frequently than suggested by the guidelines to prevent or treat PICM.

We have used the following devices for the following indications:

1. A single-chamber pacemaker (VVI) with an HBP lead in case of permanent AF with bradycardia and normal or wide QRS complex.
2. A dual-chamber pacemaker (DDD) in patients with sick sinus syndrome and a first-degree AV block and patients with a sinus rhythm and a high degree of or complete AV block—with an atrial lead and HBP lead; in patients with AF when RVP was applied as a backup—with an HBP lead in the atrial channel and ventricular lead in the right ventricle (RV).
3. A cardiac resynchronization therapy pacemaker (CRT-P) in patients with CRT indications following guidelines or in patients requiring RV backup pacing, with an HBP lead connected to the LV channel.
4. A dual-chamber implantable cardioverter-defibrillator (ICD) with a high-voltage lead in the RV and an HBP lead in the atrial channel in patients with permanent AF and indication for ICD therapy.
5. A cardiac resynchronization therapy defibrillator (CRT-D) in patients with CRT and ICD indications following guidelines, where an HBP lead was connected to the LV channel.

### 2.2. Implantation Procedure and Definition of Periprocedural Success

Antibiotic prophylaxis and Perioperative infection prevention:

1. Administration of intravenous, pre-operative antibiotic prophylaxis within 30–60 min of skin incision.
2. Skin antisepsis is performed using chlorhexidine.
3. Sterile environment, typical for surgical procedures.

The SelectSecure pacing lead (model 3830, Medtronic Inc., Minneapolis, MN, USA) was used for mapping and pacing in all cases, as previously described [14]. The lead was delivered with a fixed-shape (C315HIS, Medtronic) or deflectable (C304; Medtronic) catheter. A Medtronic pacing system analyzer (model 2290) or an electrophysiological recording system (WorkMate Claris, Abbott, Sylmar) was used for HB potential recording.

We used the procedural success definition as follows:

1. HB capture at the site with a sharp near-field His signal by QRS morphology and transition: selective HBP (s-HBP), non-selective HBP (ns-HBP), or myocardial capture. Programmed stimulation was used when QRS morphology and transition were insufficient [15].
2. Pacing capture threshold: <3.5 V @ 1.0 ms in patients with indications for CRT or <2.0 V @ 1.0 ms in patients with bradycardia indications.
3. Sensed R-wave of >1.5–2 mV without atrial oversensing.
4. Narrowing of the native QRS by more than 20% or intraventricular conduction disturbances (IVCD) correction in patients with IVCD or an RV-paced rhythm.
5. RV backup pacing was used in patients with an HV interval ≥ 70 ms or with an infra-nodal conduction block during HBP > 120/min. In patients with complete or high-degree AV block, the use of RV backup pacing was left to implanting at the physician’s discretion.

In patients with CRT indications and with unsuccessful HBP lead implantation, standard biventricular pacing with LV lead was applied.

### 2.3. Outcome Definition

Mortality and complications during F/U were used as end-points during hospitalization and for each F/U point (one month, six months, 12 months, and 24 months). The severity of complications was assessed after implantation during hospitalization, scheduled or unscheduled outpatient visits, or eventual emergency hospitalizations. During each visit, qualified medical staff assessed all clinical data, potential technical devices, or procedure-related complications. Complications were assessed separately in two periods, early (up to 30 days) and late (up to 24 months) after the procedure.

#### 2.3.1. Early Complications

Early complications during hospitalization and after discharge are characterized in Table 1.

#### 2.3.2. Late Complications

Late complications are shown in Table 2.

All complications were divided into major and minor complications. Major complications required an invasive treatment approach and were associated with a higher risk of infection. Major complications included pneumothorax requiring chest tube placement, cardiac perforation, pocket hematoma requiring surgical debridement, device pocket infectious and endocarditis, and lead dysfunction requiring reoperation. Minor complications included pocket hematoma or decubitus with conservative treatment and lead dysfunction manageable with device reprogramming [19].

### 2.4. Statistical Analysis

Continuous data were presented as mean ± SD. The normality of the data distribution was confirmed using the Kolmogorov–Smirnov test. Categorical data were expressed as counts and percentages. Occurrence rates were compared using the Chi-square test.

Univariate analyses of survival free from the composite end-point of all-cause mortality or any major complication after ICD implantation and after the upgrade procedure were performed using the Kaplan–Meier estimator with the log-rank test. Multivariate survival analysis for the same composite end-point was performed using the Cox regression full model looking for significant predictors and the stepwise method; these last were used to determine independent risk factors. The following candidate variables were used: age, male sex, the use of a high-voltage lead, LVEF, tricuspid annular plane systolic excursion (TAPSE), the left atrial area, a bundle branch block, upgrade procedure, New York Heart Association (NYHA) class, and creatinine level. The receiver operating characteristic (ROC) curve analysis determined the cut-off value if the independent predictor was a continuous variable. The area under the curve (AUC) and optimal criterion value with appropriate sensitivity and specificity for the tested variable were determined considering the cost of a false positive equaling one and the cost of a false negative equaling three.

The general criterion for statistical significance was set at *p* < 0.05. All analyses were performed by a university lecturer in medical statistics (KSG). MedCalc^®^ Statistical Software version 22.003 (MedCalc Software Ltd., Ostend, Belgium; https://www.medcalc.org, accessed on 10 February 2024; and SigmaStat version 4.0 (Systat Software, Inc., San Jose, CA, USA) were used.

The Local Bioethics Committee at the Medical University of Silesia in Katowice approved the prospective registry protocol as an investigator-initiated observational study (KatCSP-REG) (KNW/0022/KB/17/18).

## 3. Results

Successful HBP was achieved in 252 patients (68%), with an increasing success rate during consecutive years: 57% in 2015–2016 and 73% in 2017–2019. The complete F/U was obtained as follows: 30 days in 232/252 (91%) patients, six months in 224/252 (88%) patients, 12 months in 201/252 (79%) patients, and 24 months in 174/252 (69%) patients. The baseline characteristics, comorbidities and pharmacotherapy of patients are shown in Table 3. The two groups (ICD/CRT-D and PM/CRT-P) differed in the number of men in the study group, echocardiography parameters [left ventricle end-diastolic diameter (LVEDD), left ventricle end-systolic diameter (LVESD), left ventricle end-diastolic volume (LVEDV), left ventricle end-systolic volume (LVESV), and left ventricle ejection fraction (LVEF)], and NYHA class (Table 4).

**Figure 1:**
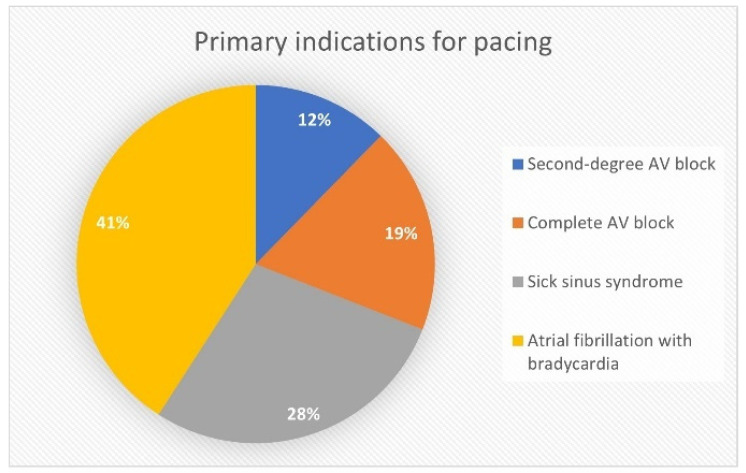
Primary indications for pacing.

**Figure 2:**
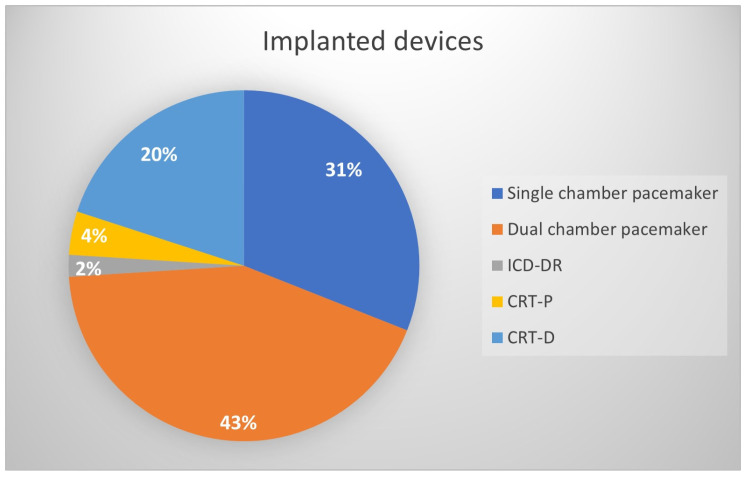
Implanted devices.

The primary indications for permanent pacing were as follows: second-degree AV block in 31 patients (12.3%), complete AV block in 47 patients (18.7%), sinus node dysfunction in 71 patients (28.2%), and AF with bradycardia in 103 patients (40.9%) (Figure 1). Predominantly implanted systems were dual-chamber devices, 108 (42.9%) (Figure 2). A total of 202 (80.2%) devices were implanted de novo, and 50 (19.8%) were implanted as a system upgrade. Procedural data are shown in Table A2 (Appendix B section).

### 3.1. Mortality and Complications

There were no deaths during the first 30-day period. A total of 26 patients (10.3%) died within 24 months. Six- and twelve-month mortality rates were 3.2% (*n* = 8) and 4.8% (*n* = 12), respectively. There was no procedure-related death; 3.2% of patients (*n* = 8) died from other cardiovascular causes. In six (2.4%) cases, the cause of death could not be determined.

**Figure 3:**
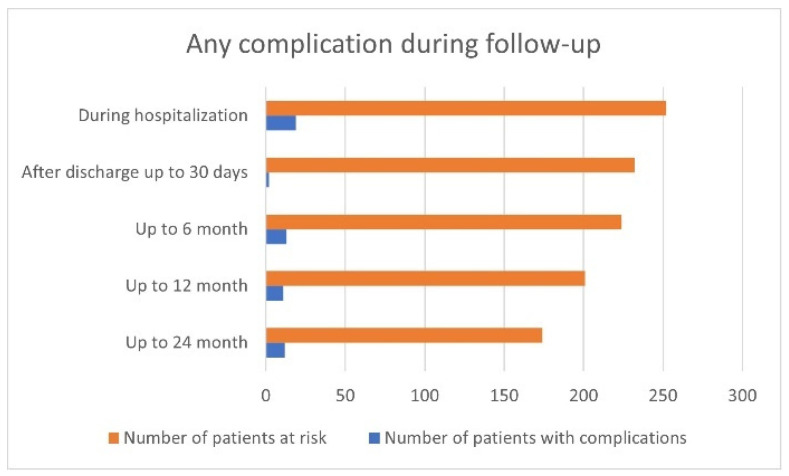
The distribution of complication frequency (both major and minor) during subsequent follow-up stages.

Complications were found in 7.5% of patients (19/252) during primary hospitalization and in 0.9% (2/232), 5.8% (13/224), 5.5% (11/201), and 6.9% (12/174) at 30 days, 6 months, 12 months, and 24 months, respectively (Figure 3). Up to 24 months F/U, 23 major complications were reported: nine within 30 days, five in 6 months, five in 12 months, and four in 24 months of observation.

### 3.2. During Hospitalization and Short-Term up to 30 Days Follow-Up

Within 30 days after the procedure, 21 complications (36.9% of all complications) occurred in 19 patients, with more than one in two patients. In seven patients, complications occurred during the upgrade procedure. Most complications (90.5%) occurred during in-hospital observation. There were no infectious complications during short-term F/U (Table 5).

As a noninfectious complication associated with the device pocket, pocket hematoma occurred only before discharge in six cases. Five patients were treated conservatively and one required a surgical intervention. No similar complication was observed at the 30-day post-discharge F/U.

We observed one lead dysfunction manageable with device reprogramming during in-hospital observation. The pacing threshold increased from 1.9 V at 1.0 ms to 3.0 V at 1.0 ms, although it remained stable during F/U. In the 30-day F/U after discharge, HBP lead atrial oversensing was observed in two patients, amenable to reprogramming.

The HBP lead was dislodged twice; in both cases, the HBP lead was reimplanted successfully. Five dislodgements were related to non-HBP leads, with four atrial and one RV lead dislodgement. All lead dysfunctions requiring repositioning were present only in the in-hospital observation.

Four cases of traumatic complications were noted. They all resulted from subclavian vein puncture—pneumothorax in two patients, subclavian vein thrombosis in one patient, and hemoptysis, probably due to bronchial puncture. No additional complication was observed at the 30-day F/U.

### 3.3. Long-Term, up to 24 Months after Implantation Follow-Up

There were 36 complications (63.2%) in the long-term F/U in 31 patients: 13 during the first six months, 11 during the subsequent six months, and 12 in the remaining 24-month F/U. Five patients in long-term observation had more than one complication. In 11 cases, complications occurred after the device upgrade procedure (Table 6).

There was only one pocket-related noninfectious complication during the six-month observation: the patient was diagnosed with a hematoma with the formation of a skin fistula without an inflammatory process. Hematoma draining and negative-pressure wound therapy were performed successfully.

Nineteen lead dysfunction complications manageable with device reprogramming were reported: six in the six-month F/U, another six in the 12-month F/U, and seven in the 24-month F/U. During the six-month F/U, oversensing of pectoral myopotentials was found in one case due to unipolar sensing configuration (with bipolar sensing, occasional R-wave undersensing was observed). Pectoral muscle stimulation occurred in one patient with HBP unipolar pacing. In both cases, changing the sensing or pacing polarity to bipolar resolved the issue. There was a significant increase in the pacing threshold in the remaining four cases, but the values subsequently remained stable and were acceptable.

In the 12-month F/U period, HBP leads sensing problems were detected in two patients, one R-wave undersensing and one T-wave oversensing. In both cases, it was necessary to change the sensitivity settings. In one case, premature ventricular contraction oversensing on the atrial channel was present, and the device reprogramming pacing mode resolved the problem. A significant increase in the pacing threshold was found in two cases. Adjustments of the pacing amplitude were sufficient to maintain adequate and safe HBP capture.

At the 24-month F/U, HBP lead oversensing was observed in three patients. In cases with pectoral myopotential oversensing and T-wave oversensing, changing the sensing configuration from unipolar to bipolar resolved the sensing issues. In a patient with a DDD pacemaker and RV backup lead, HBP lead far-field R-wave oversensing was present and was corrected by reprogramming the device to DVI pacing. There were three cases with an increase in the HBP threshold. In two CRT devices, a significantly increased pacing threshold of HBP lead connected to the LV channel was reported (in one case, over 4.0 V at 1.0 ms and 4.5 V at 1.0 ms in the second one). In both cases, switching off the LV lead was necessary. These patients were not ultimately referred for coronary sinus lead reimplantation. The decision regarding the management strategy was based on individual risk-benefit analysis as part of a shared decision-making process with the patient.

During the six months of F/U, four malfunctioning leads required reoperation, and in three cases, it was due to HBP lead dislodgement. Successful HBP was obtained again in two patients, and RV septal pacing was applied in another. In the fourth case, atrial lead dislodgement was resolved through lead repositioning. In the 12-month F/U, there were four HB lead exit blocks and one RV defibrillation lead dislodgment. In every case, leads were reimplanted, and physiological pacing was reapplied in two patients. RV septal pacing was used in two cases, and an LV epicardial pacing lead was implanted in another. In the 24-month F/U, each case of lead dysfunction was associated with HB lead. There were three exit blocks and one dislodgment. In all of them, RV septal pacing was used instead of physiological stimulation. A total of three infectious complications were recorded. At the six-month F/U, there was one pocket infection without a systemic inflammatory process and one lead-associated endocarditis. In both cases, the pacing system was removed. During the 24-month F/U, there was one patient with lead-associated endocarditis. While extraction of the CIED was necessary, reimplantation was not required.

### 3.4. Prediction of the Composite End-Point of All-Cause Mortality or Any Major Complication

**Figure 4:**
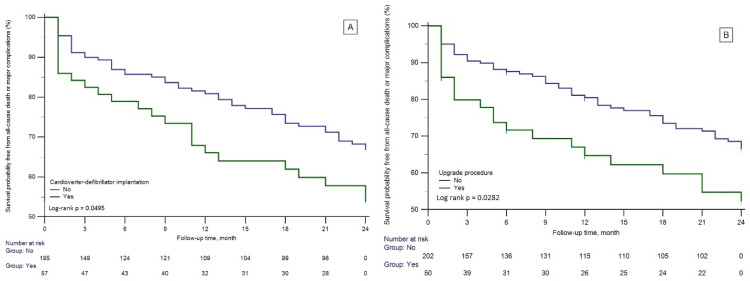
Kaplan–Meier curves of survival probability free from all-cause death or major complications depending on the cardioverter-defibrillator implantation (**A**) and depending on the upgrade procedure (**B**).

An ICD was implanted in 57 patients, 22.6% of the study group. The Kaplan–Meier estimate of the probability of survival free from the composite end-point of all-cause mortality or any major complication in 24 months of F/U of ICD-implanted patients was lower than pacemaker-implanted ones, with a hazard ratio of 1.7142 with a 95% confidence interval of 1.012 to 2.9350, log-rank *p* = 0.0495 (Figure 4A). The upgrade procedure was performed in 50 patients, 19.8% of the study group. The probability of survival free from the composite end-point of upgraded patients was lower compared to other patients, with a hazard ratio of 1.9121 an a 95% confidence interval of 1.0716 to 3.4121, log-rank *p* = 0.0282 (Figure 4B).

Using a Cox regression analysis, the lower LVEF is the only independent predictor of the composite end-point (Table 7). The cut-off value of the LVEF, for which the probability of an event occurrence increases, was ≤34% (AUC 0.609, *p* = 0.0195, with a sensitivity of 34.7% and a specificity of 83.3%). The occurrence of events was higher in patients with an LVEF ≤ 34%, *p* = 0.0247.

## 4. Discussion

The HBP implant procedure utilizes standard surgical approaches but also requires specific and sensing issues [16,17,20].

In our study, in short-term F/U, complications related directly to the HB lead occurred in 2.0% of patients, and “electrical” complications occured in only 1.2%. This value is lower than the reported threshold increase of >1 V in 28% of patients for intermediate-term F/U [16], but the HBP threshold rise may occur up to one year after implant [21]. Modifying the standard implant technique and using a dedicated sheath did not translate to an additional risk of periprocedural complications, as the risk of pocket hematoma, lead dislodgment, and traumatic complications was similar to standard pacemaker implantation [10,19]. In long-term F/U, HBP-related complications were most common. A threshold increase of >1 V or a loss of HB capture occurred in 9.2% of patients, and the incidence of capture issues was lower than previously reported [16,22], which in part may result from the amount of lead slack used in our center, as the HB lead slack has an impact on threshold stability [21]. The sensing issues (3.4%) with the most common R-wave undersensing were more common than previously reported [16,18], and this may be due to the infrequent use of RV backup lead in our center. The incidence of pocket infection after six months (0.9%) and 24 months (0.6%) was similar to standard pacemaker implantation [10,19], although the HB lead implant procedure may be longer than the RV lead implant. Overall, we had a 20.7% complication rate, with 8.1% major complications in long-term F/U, and these results are consistent with the results of the FOLLOWPACE study [10] for standard pacing.

Both in short-term and long-term F/U, the complication rate was higher for upgrade procedures than for de novo implants, and these results are consistent with previously reported outcomes of procedures with lead addition [23]. There was no in-hospital and 30-day mortality in our study. According to other authors, the all-cause mortality rate is up to 1.3% and 1.4% (in-hospital and 30-day mortality), respectively [19,24]. We observed six- and 12-month mortality rates of 3.2% and 4.8%. Earlier pre-CSP publications reported a long-term mortality rate from 3.2% to 6.2% [19,25]. We found a 24-month, all-cause mortality rate of 10.3%. The MADIT II trial reported a mortality rate of 14.2% at an average FU of 20 months [26].

A primary line of division of our study group into subpopulations seems to use or not use devices with and without ICD. The participants from the former subgroup had larger left ventricular dimensions and volumes, lower LVEF, and a higher NYHA functional class. When the survival of both subgroups free of the applied composite end-point was compared by the univariate Kaplan–Meier estimator, it was worse in the ICD subgroup. Similarly, an upgrade procedure worsened the survival rate if assessed using a univariate approach. The composite end-point was reached earlier in these patients.

However, according to our data, despite significant differences between subgroups with and without ICD implanted, high-voltage device implantation does not affect survival in the 24-month perspective using the multivariate Cox approach adjusted to age and sex. Recent data on long-term survival comparison between CRT pacemakers and CRT defibrillator recipients show similar results [27]. Although they concern patients with biventricular pacing, comparing HBP with and without ICD therapy can be considered an analogy between a CRT pacemaker and a CRT defibrillator.

Also, there is no general agreement on the hypothetical worse survival of patients after the upgrade procedure compared to patients who received similar, more complex stimulation de novo. Data comparing the clinical response and survival in patients undergoing CRT upgrade and de novo CRT implantation showed significantly worse effects in the former group [28]. In another retrospective analysis, patients subjected to an upgrade procedure had higher all-cause mortality than de novo implanted patients. The more significant number of concomitant diseases in the upgraded group was raised as a significant contributing factor [29]. On the other hand, a reasonably large meta-analysis of de novo implantation vs. upgrade cardiac resynchronization therapy indicates that all-cause mortality was similar after the CRT upgrade compared to de novo implantations [30]. Also, in our group of patients who were paced biventricularly in the His bundle part of CSP, in our results from the multivariate survival analysis, an upgrade procedure is not an independent predictor of survival.

The Cox regression full model approach revealed male sex and a lower LVEF as significant predictors of the composite end-point of all-cause mortality or any major complication we found in the 24-month follow-up. Similar data regarding the male gender as a predictor of mortality were presented earlier [10,24,31]. However, using a stepwise approach, the lower LVEF is the only independent predictor. It is consistent with previous reports [32]. Simultaneously, other indicators of heart failure, right ventricle function, and functional class were not strong enough to be independent predictors. It is worth noting that the cut-off value of LVEF associated with worse survival that we found is almost identical to the LVEF value for which European and United States guidelines for the management of patients with ventricular arrhythmias and the prevention of sudden cardiac death recommend the use ICD for the primary prevention of this last.

Our success rate is lower than previously reported. That is probably because our data represent the success rate from the first implantation and include a multi-operator learning curve. As presented by Keene et al. [20], the success rate improves after completion of 40 cases.

## 5. Conclusions

This single-center study reported a low risk of all-cause mortality and overall and major complications associated with HBP at short-term FU. However, we observed a higher but acceptable risk of major complications under long-term observation. A lower LVEF was an independent predictor of the composite end-point of all-cause mortality or any major complication at the 24-month F/U. Our data confirmed that HBP is a technique that should be considered a feasible and safe alternative to RV or classical biventricular pacing.

## 6. Study Limitations

Our report was designed to be a maximally detailed single-center report of HBP complications. Patients with successful HPP implantation were followed up in more detail than in the standard postoperative follow-up procedure. There was no possibility of creating a formal control group because we do not have the required detailed data on the course of complications in patients with a classical method of implantation. However, in Appendix A, we present data on the follow-up of patients in whom HBP could not be performed due to periprocedural problems.

It should be noted that data come from a prospective registry but from a single-center, which is why there is a limited number of enrolled patients. The frequency of the HBP procedure significantly decreased in our center in favor of performing left bundle branch pacing. In our site during the “only HBP era”, we had several operators with varying degrees of experience in HBP, and the learning curve was not analyzed. Another limitation is the F/U duration of only 24 months. Therefore, an extended observation period would be valuable. Large, multicenter, high-volume observations are needed to assess HBP therapy safety comprehensively.

## Figures and Tables

**Table 1:** Early complications.

Traumatic, Periprocedural Complications	Device Pocket Noninfectious Complications	Infectious Complications	Lead Dysfunction Manageable with Device Reprogramming	Lead Dysfunction Requiring Reoperation
Pneumothorax Pericardial effusion and/or cardiac tamponade Subclavian vein thrombosis	Pocket hematoma Pocket decubitus	Pocket local infection Systemic infection	Threshold rise ≥1 V from an implant ^1^ R-wave undersensing Atrial oversensing T-wave oversensing Phrenic nerve stimulation	Capture or sensing abnormalities not manageable with reprogramming Lead dislodgement Lead fracture or insulation break

^1^ according to other authors [16,17,18].

**Table 2:** Late complications.

Device Pocket Noninfectious Complications	Infectious Complications	Lead Dysfunction Manageable with Device Reprogramming	Lead Dysfunction Requiring Reoperation
Pocket hematoma Pocket decubitus	Device pocket infectious complications and endocarditis	Threshold rise ≥1 V from an implant ^1^ R-wave undersensing Atrial oversensing T-wave oversensing Phrenic nerve stimulation	Capture or sensing abnormalities not manageable with reprogramming Lead dislodgement Lead fracture or insulation break

^1^ according to other authors [16,17,18].

**Table 3:** Baseline characteristics, comorbidities, and pharmacotherapy.

Baseline Characteristics	
Males	171 (48%)
Age (years)	71 ± 11
NYHA class	1.9 ± 0.8
**Echocardiography Parameters**	
LVEDD [mm]	53.8 ± 8.7
LVESD [mm]	38.7 ± 10.6
LVEDV [mL]	148.1 ± 62.6
LVESV [mL]	80.7 ± 52.7
LVEF [%]	49.0 ± 14.1
**Comorbidities**	
Atrial fibrillation/flutter	139 (55%)
Coronary artery disease, *n*; %	124; 49%
Prior myocardial infarction *n*; %	56; 22%
Prior percutaneous coronary intervention, *n*; %	69; 27%
Prior coronary bypass grafting, *n*; %	41; 16%
Stroke, *n*; %	36; 14%
Arterial hypertension, *n*; %	193; 77%
Diabetes, *n*; %	83; 33%
Renal insufficiency, eGFR < 60 mL/min/1.73 m^2^, *n*; %	51; 20%
Hyperlipidemia, *n*; %	168; 67%
**Pharmacotherapy**	
Use of diuretic, *n*; %	125; 50%
Use of ACEI/ARB, *n*; %	177; 70%
Use of beta-blocker, *n*; %	211; 84%
Use of aldosterone receptor blocker, *n*; %	126; 50%
Use of calcium-channel blocker, *n*; %	69; 27%
Use of amiodarone, *n*; %	21; 8%
Use of digitalis, *n*; %	34; 13%
Use of statins, *n*; %	168; 67%
Use of anticoagulants, *n*; %	160; 63%
Use of ivabradine, *n*; %	3; 1%

NYHA—New York Heart Association Functional Classification, LVEDD—left ventricle end-diastolic diameter, LVEDV—left ventricle end-diastolic volume, LVEF—left ventricle ejection fraction, LVESD—left ventricle end-systolic diameter, LVESV—left ventricle end-systolic volume, PM—pacemaker, NYHA—New York Heart Association Functional Classification, eGFR—estimated glomerular filtration rate, ACEI/ARB—angiotensin-converting-enzyme inhibitors/angiotensin II receptor blockers.

**Table 4:** ICD/CRT-D and PM/CRT-P group comparison.

	ICD/CRT-D	PM/CRT-P	*p*
Males	45 (79%)	126 (65%)	*p* = 0.0419
Age (years)	67.9 ± 8.9	72.5 ± 11.4	*p* = 0.2128
LVEDD [mm]	62.1 ± 8.3	51.1 ± 8.0	*p* < 0.0001
LVESD [mm]	49.6 ± 9.6	35.0 ± 9.1	*p*< 0.0001
LVEDV [mL]	217 ± 75.2	121.1 ± 49.5	*p* = 0.0322
LVESV [mL]	136.2 ± 69.1	57.8 ± 33.0	*p* = 0.0003
LVEF [%]	31.5 ± 8.7	54.1 ± 11.0	*p* < 0.0001
NYHA class	2.4 ± 0.73	1.8 ± 0.7	*p* < 0.0001
Atrial fibrillation/flutter	26 (46%)	112 (58%)	*p* = 0.2180

CRT-D—implantable cardiac resynchronization therapy defibrillator, CRT-P—implantable cardiac resynchronization therapy pacemaker, ICD—implantable cardioverter-defibrillator, NYHA—New York Heart Association Functional Classification, LVEDD—left ventricle end-diastolic diameter, LVEDV—left ventricle end-diastolic volume, LVEF—left ventricle ejection fraction, LVESD—left ventricle end-systolic diameter, LVESV—left ventricle end-systolic volume, PM—pacemaker.

**Table 5:** Complications occurring in hospital and during 30-day follow-up.

	In Hospital *n* = 252	Device Type (n)	System Upgrading *n* = 50	After Discharge *n* = 232	Device Type (n)	System Upgrading *n* = 47
Device-pocket noninfectious complications	6/252; 2.4%	VVI (1) DDD (1) CRT-D (2) ICD-DR (2)	3/50; 6.0%	0	0	0
Lead dysfunction manageable with device reprogramming	1/252; 0.4%	CRT-D (1)	0	2/232; 0.9%	VVI (1) ICD-DR (1)	0
Lead dysfunction requiring reoperation	7/252; 2.8%	DDD (3) CRT-D (3) CRT-P (1)	2/50; 4.0%	0	0	0
Traumatic, periprocedural complications	5/252; 2.0%	VVVI (1) DDD (2) CRT-D (2)	2/50; 4.0%	0	0	0
Infectious complications	0	0	0	0	0	0

VVI—single chamber pacemaker, DDD—dual chamber pacemaker, ICD-DR—dual chamber implantable cardioverter-defibrillator, CRT-P—implantable cardiac resynchronization therapy pacemaker, CRT-D—implantable cardiac resynchronization therapy defibrillator.

**Table 6:** Complications during long-term follow-up.

	6-Month *n* = 224	Device Type (n)	System Up-Grading *n* = 44	12-Month *n* = 201	Device Type (n)	System Up-Grading *n* = 38	24-Month *n* = 174	Device Type (n)	System Up-Grading *n* = 32
Device-pocket noninfectious complications	1/224; 0.5%	ICD-DR (1)	1/44; 2.3%	0	0	0	0	0	0
Lead dysfunction manageable with device reprogramming	6/224; 2.7%	VVI (3) DDD (2) CRT-P (1)	0	6/201; 3.0%	VVI (2) DDD (1) CRT-D (3)	2/38; 5.3%	7/174; 4.0%	VVI (1) DDD (1) CRT-P (3) CRT-D (2)	2/32; 6.3%
Lead dysfunction requiring reoperation	4/224; 1.8%	DDD (2) CRT-D (2)	2/44; 4.6%	5/201; 2.5%	VVI (1) DDD (1) CRT-D (3)	1/38; 2.6%	4/174; 2.3%	VVI (1) DDD (2) CRT-D (1)	1/32 3.1%
Device pocket infectious complications and endocarditis	2/224; 0.9%	ICD-DR (1) DDD (1)	2/44; 4.6%	0	0		1/174; 0.6%	ICD-DR-1	0

VVI—single chamber pacemaker, DDD—dual chamber pacemaker, ICD-DR-Dual chamber implantable cardioverter-defibrillator, CRT-P—implantable cardiac resynchronization therapy pacemaker, CRT-D—implantable cardiac resynchronization therapy defibrillator.

**Table 7:** Prediction of the composite end-point of all-cause mortality or any major complication in 24-month follow-up.

Predictor	Full Model			Stepwise Regression		
	HR	95%CI	*p*	HR	95%CI	*p*
Age (years)	1.0150	0.9831–1.0479	0.3613	-	-	-
Sex (male)	2.1155	0.9392–4.7653	0.0405	-	-	-
ICD	0.6611	0.2750–1.5895	0.3552	-	-	-
LVEF (%)	0.9811	0.6644–2.8542	0.0041	0.9793	0.9594–0.9996	0.0454
TAPSE (mm)	0.9626	0.8933–1.0373	0.3172	-	-	-
Left atrial area (cm^2^)	0.9715	0.6644–2.8542	0.3896	-	-	-
Bundle branch block	1.3770	0.4242–2.9421	0.3896	-	-	-
Upgrade procedure	1.4797	0.7064–3.0993	0.2989	-	-	-
NYHA class	0.9665	0.5900–1.5833	0.8925	-	-	-
Creatinine level (mg/dL)	0.8861	0.3476–2.2586	0.8000	-	-	-

ICD—cardioverter-defibrillator usage, LVEF—left ventricle ejection fraction, NYHA—New York Heart Association Functional Classification, TAPSE—tricuspid annular plane systolic excursion.

**Figure A1:**
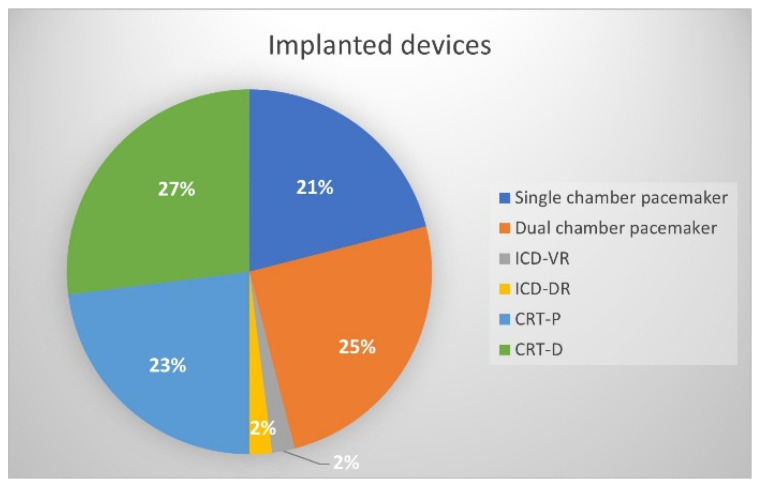
Implanted devices in control group.

## Data Availability

The data presented in this study are available on request from the corresponding author.

## Appendix A

### Appendix A.1. Control Group

The control group presents data from 121 patients in whom HBP could not be performed. Predominantly implanted systems were CRT-D (27%) (Figure A1).

### Appendix A.2. Mortality and Complications

There were no deaths during the first 30-day period. A total of 11 patients (9.1%) died within 24 months. Six- and twelve-month mortality rates were 1.7% (*n* = 2) and 0.8% (*n* = 1), respectively.

Complications were found in 9.9% of patients during primary hospitalization and 4.1% in long-term observation. Up to 24 months F/U, seven major complications were reported: six within 30 days, one in six months observation, and there were no major complications at the 12- and 24-month F/U. Complication data are shown in Table A1.

**Table A1:** Early and late complications in the control group.

	In-Hospital and during 30-Day Follow-Up	Device Type (n)	Long-Term Follow-Up	Device Type (n)
Device-pocket noninfectious complications	4.1%	CRT-P (3) CRT-D (2)	0.8%	CRT-D (1)
Lead dysfunction manageable with device reprogramming	0.8%	DDD (1)	3.3%	DDD (1) CRT-P (2) CRT-D (1)
Lead dysfunction requiring reoperation	2.5%	VVI (1) DDD (1) CRT-P (1)	0	0
Traumatic, periprocedural complications	2.5%	DDD (2) CRT-P (1)	0	0
Infectious complications	0	0	0	0

VVI—single chamber pacemaker, DDD—dual chamber pacemaker, ICD-DR—dual chamber implantable cardioverter-defibrillator, CRT-P—implantable cardiac resynchronization therapy pacemaker, CRT-D—implantable cardiac resynchronization therapy defibrillator.

## Appendix B

**Table A2:** Procedural data.

	VVI	DDD	ICD-DR	CRT-P	CRT-D
Implant time [min]	57.8 ± 28.1	72.5 ± 37.8	96.0 ± 22.7	84.0 ± 38.9	82.1 ± 51.7
Fluoroscopy time [min]	11.4 ± 12.3	13.3 ± 10.8	13.4 ± 7.2	20.6 ± 15.8	14.9 ± 14.5
Radiation dose [mGy]	49.3 ± 61.4	66.0 ± 109.2	56.9 ± 27.2	74.6 ± 82.8	92.1 ± 135.2

VVI—single chamber pacemaker, DDD—dual chamber pacemaker, ICD-DR—Dual chamber implantable cardioverter-defibrillator, CRT-P—implantable cardiac resynchronization therapy pacemaker, CRT-D—implantable cardiac resynchronization therapy defibrillator.
